# Implementation of Copy Number Variations-Based Diagnostics in Morphologically Challenging *EWSR1/FUS::NFATC2* Neoplasms of the Bone and Soft Tissue

**DOI:** 10.3390/ijms232416196

**Published:** 2022-12-19

**Authors:** Iva Brcic, Susanne Scheipl, Marko Bergovec, Andreas Leithner, Joanna Szkandera, Karl Sotlar, Arnold Suda, Maria Anna Smolle, Tanja Kraus, Andrew Eric Rosenberg, Bernadette Liegl-Atzwanger, Jasminka Igrec

**Affiliations:** 1 Diagnostic and Research Institute of Pathology, Medical University of Graz, Comprehensive Cancer Centre, 8010 Graz, Austria; 2 Department of Orthopedics and Trauma, Medical University of Graz, Comprehensive Cancer Centre, 8010 Graz, Austria; 3 Division of Clinical Oncology, Department of Medicine, Medical University of Graz, Comprehensive Cancer Centre, 8010 Graz, Austria; 4 Institute of Pathology, Paracelsus Medical University Salzburg, 5020 Salzburg, Austria; 5 Department of Orthopedics and Trauma Surgery, AUVA Trauma Center Salzburg, 5010 Salzburg, Austria; 6 Department of Pathology and Laboratory Medicine, University of Miami, Miami, FL 33136, USA; 7 Division of General Radiology, Department of Radiology, Medical University of Graz, Comprehensive Cancer Centre, 8010 Graz, Austria

**Keywords:** *EWSR1*, *FUS*, *NFATC2*, simple bone cyst, copy number variations, alterations, sarcoma

## Abstract

In the last decade, new tumor entities have been described, including *EWSR1/FUS::NFATC2*-rearranged neoplasms of different biologic behavior. To gain further insights into the behavior of these tumors, we analyzed a spectrum of *EWSR1/FUS::NFATC2*-rearranged neoplasms and discuss their key diagnostic and molecular features in relation to their prognosis. We report five patients with *EWSR1/FUS::NFATC2*-rearranged neoplasms, including one simple bone cyst (SBC), two complex cystic bone lesions lacking morphological characteristics of SBC, and two sarcomas. In three cases, fluorescence in situ hybridization (FISH) and in all cases copy number variation (CNV) profiling and fusion analyses were performed. All patients were male, three cystic lesions occurred in children (aged 10, 14, and 17 years), and two sarcomas in adults (69 and 39 years). Fusion analysis revealed two *FUS::NFATC2* rearrangements in two cystic lesions and three *EWSR1::NFATC2* rearrangements in one complex cystic lesion and two sarcomas. *EWSR1* FISH revealed tumor cells with break-apart signal without amplification in one complex cystic lesion and *EWSR1* amplification in both sarcomas was documented. CNV analysis showed simple karyotypes in all cystic lesions, while more complex karyotypes were found in *NFATC2*-rearranged sarcomas. Our study supports and expands previously reported molecular findings of *EWSR1/FUS::NFATC2*-rearranged neoplasms. The study highlights the importance of combining radiology and morphologic features with molecular aberrations. The use of additional molecular methods, such as CNV and FISH in the routine diagnostic workup, can be crucial in providing a correct diagnosis and avoiding overtreatment.

## 1. Introduction

The WHO classification of primary bone and soft tissue neoplasms has been constantly refined over the past two decades. The modifications of the classification scheme have been significantly accelerated by the incorporation of genetic aberrations identified by next generation sequencing (NGS) technologies. In the current fifth edition of the WHO book on *Soft Tissue and Bone Tumors*, the molecular signature of tumors has been incorporated into their definition, which underscores the importance of molecular diagnostics in the routine work-up of soft tissue and bone neoplasms [1].

Tumors with *EWSR1/FUS::NFATC2* gene fusions have a broad spectrum of morphology and biological behavior and include vascular malformation, hemangioma, epithelioid vascular neoplasms, simple bone cyst (SBC), and round cell sarcomas of bone and soft tissues [2,3,4,5,6,7,8,9,10,11,12]. To gain further insight into the behavior of these tumors, we analyzed a spectrum of *EWSR1/FUS::NFATC2*-rearranged neoplasms and discuss their radiologic, diagnostic, and molecular features in relation to their prognosis.

## 2. Results

Clinical, immunohistochemical, and molecular data are summarized in Table 1. Radiological features are outlined in Table 2.

### 2.1. A Simple Bone Cyst (Case #1)

**Figure 1:**
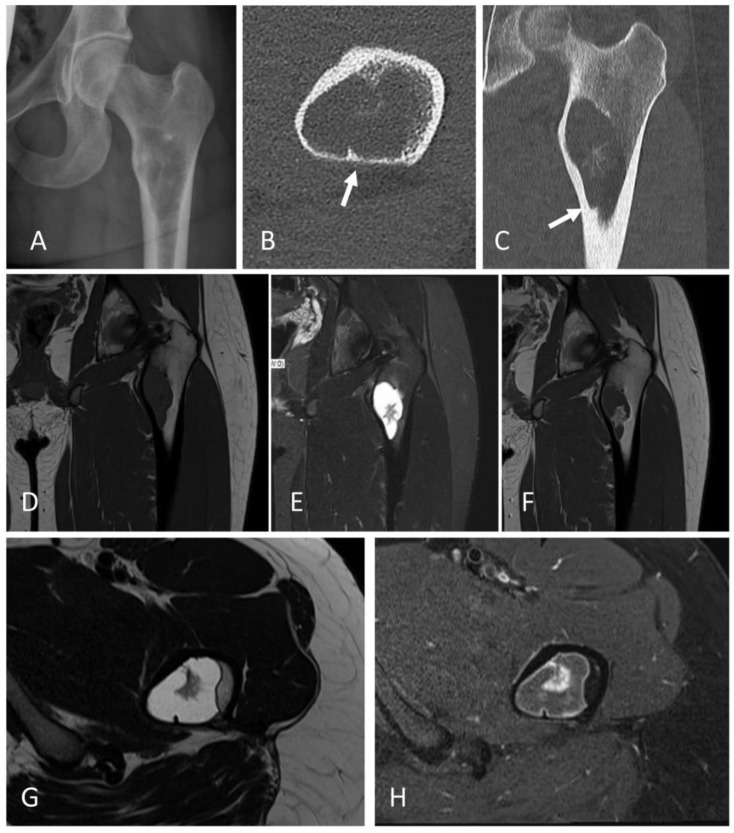
The 17-year-old boy with a lytic lesion of the proximal left femur (case #1) with a *FUS::NFATC2* fusion. (**A**) AP-radiograph; (**B**,**C**) axial and coronal CT-reformatted images show a hypodense lesion with focal central calcifications, thinning of the cortex (white arrows), and intralesional subseptations; (**D**–**H**) MRI shows that the tumor T1-weighted images have intermediate signal (**D**) and high signal on T2-weighted images (**E**,**G**) with enhancement of the cyst wall and irregular central subseptations (**F**,**H**). No bone marrow edema or periosteal reaction are present.

**Figure 2:**
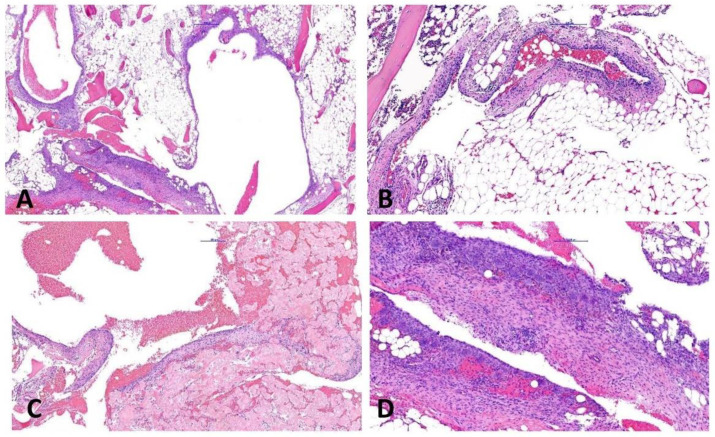
(**A**,**B**) The cystic tumor (case #1) has thin fibrous walls. (**C**,**D**) Unmineralized fibrin-like deposits of collagen are present along with plump, cytological bland, ovoid, and spindle cells with scattered osteoclast-like giant cells.

**Figure 3:**
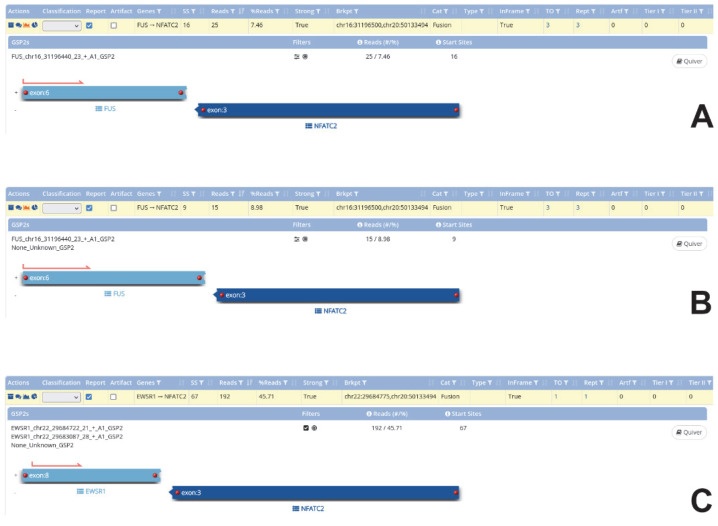
Schematic diagram of the fusion transcript for three cases. (**A**,**B**) In cases #1 and #2 *FUS::NFATC2* fusion and (**C**) in case #4 *EWSR1::NFATC2* fusion were documented. Images show the genes, exons, and locations of each fusion.

A 17-year-old male patient was admitted with pain due to an acute fracture after slipping while walking.. Radiographs revealed within the left proximal femoral diaphysis a 3.2 cm large geographic lucent lesion with a narrow zone of transition and thin septations (Figure 1A–H). The lesion was curetted and stabilized with internal fixation. Histologically, the tumor was cystic, and the cyst walls were moderately cellular and composed of scattered bland spindle to ovoid cells admixed with scattered foamy histiocytes, osteoclast-like giant cells, and unmineralized fibrin-like collagen (Figure 2A–D). Molecular analysis showed a *FUS::NFATC2* fusion (Figure 3A) and a simple/normal karyotype (Supplementary Figure S1A). During 12 months of follow-up, the patient was without symptoms and no local recurrence.

### 2.2. Complex Cystic Bone Lesions (Cases #2 & 3)

**Figure 4:**
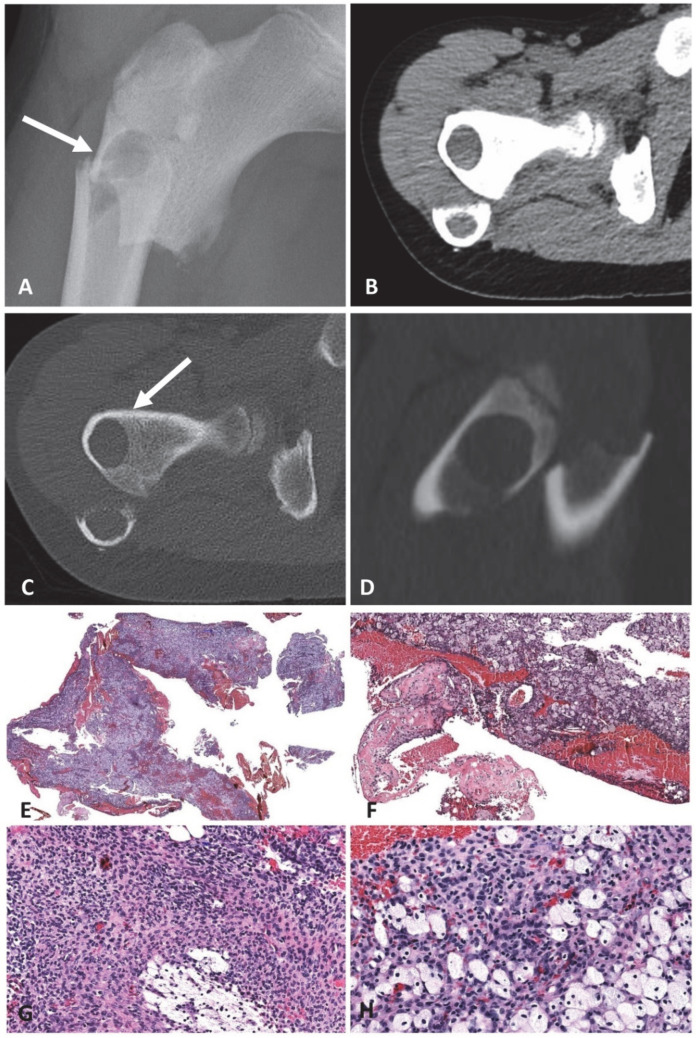
The 10-year-old-boy with a complex cystic tumor harboring a *FUS::NFATC2* fusion in the right proximal femur (case #2). (**A**) The tumor is intramedullary, eccentric, well-defined, lytic, and associated with a displaced fracture. A small incidental enostosis is present in the femoral neck. (**B**–**D**) Soft-tissue (**B**) and high-resolution bone windows CT (**C**,**D**) show a well-defined lytic lesion with a thin sclerotic margin (arrow). The tumor contains hyperdense tissue, which probably represents blood (**B**). (**E**–**H**) Histology shows a cystic lesion composed of sheets of foamy histiocytes and bland spindled and ovoid cells that have vesicular nuclei, and pale eosinophilic cytoplasm. In one area, unmineralized fibrinous, cementum-like tissue (**F**) is present.

**Figure 5:**
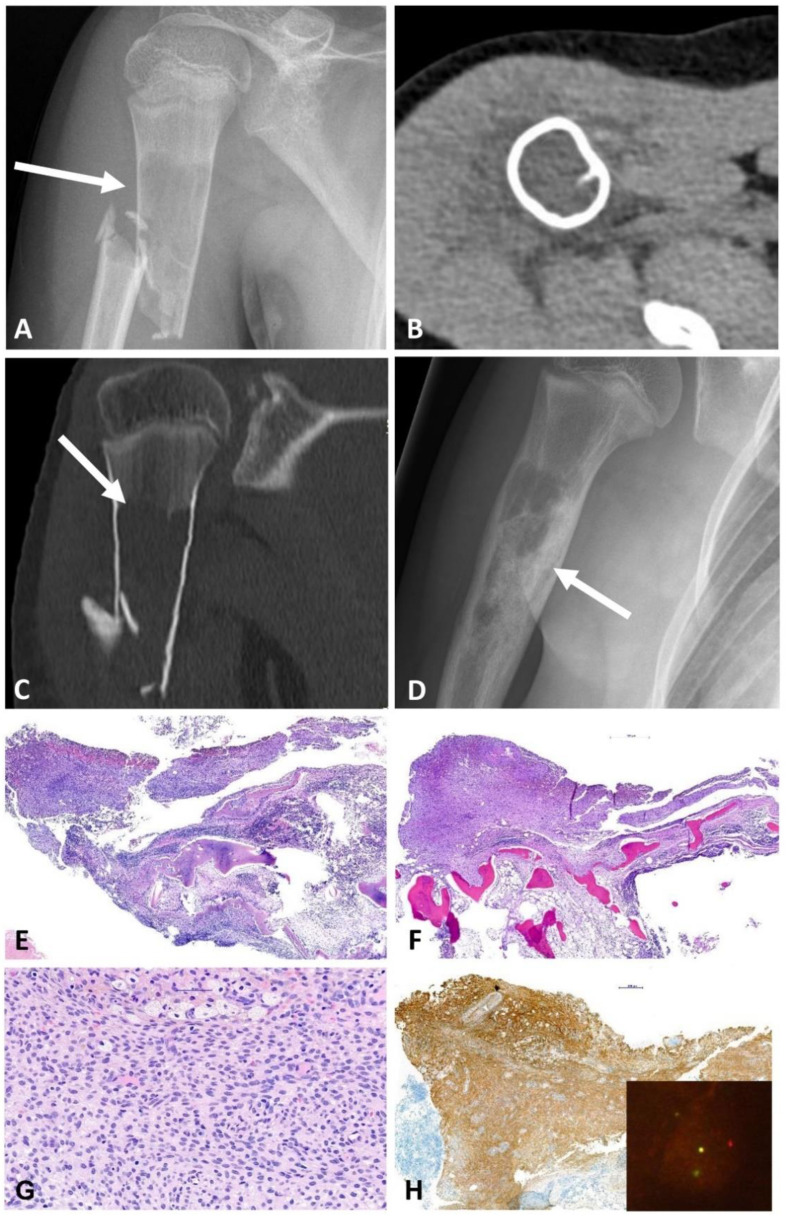
The 14-year-old-boy with a lytic lesion in the proximal humeral diaphysis (case #3) with a *EWSR1::NFATC2* fusion. (**A**) AP radiograph shows that the tumor is central, lytic, well-circumscribed (arrow) with a thin sclerotic rim and has undergone pathological fracture. Axial soft tissue-weighted CT image (**B**) shows hyperdense intracystic content, probably caused by blood. There is circumferential deep endosteal scalloping ((**C**), arrow), and the margin is distinct, but not sclerotic. (**D**) Ten months after curettage, the tumor has intralesional calcifications with a thickened cortex (arrow). (**E**–**H**) Histologically, the cyst walls are thick and composed of woven bone and a bland monotonous proliferation of spindled and ovoid cells that have vesicular nuclei, and pale eosinophilic cytoplasm; scattered histiocytes are present. (**H**) The tumor cells are positive for CD99. FISH with *EWSR1* probe reveals cells with break-apart (split) signal and no evidence of *EWSR1* amplification (inset).

Two male patients, 10 and 14 years old, presented with pain caused by spontaneous non-traumatic, comminuted, pathologic fractures that involved the right proximal femur and the right proximal humerus diaphysis, respectively. Both lesions were well-circumscribed, lytic, and expansile with endosteal scalloping. There was no cortical transgression. The tumor in the humerus had thin septations (Figure 4A–D and Figure 5A–D). The femoral lesion was 2.9 cm large, and the humeral lesion measured 6.7 cm. Both lesions were curetted, and internal fixation was placed.

Histologically, the cyst walls of both tumors were thick and composed of a bland proliferation of spindle and ovoid cells that have vesicular nuclei and pale eosinophilic cytoplasm (Figure 4E–H and Figure 5E–H). Foamy histiocytes were scattered through the neoplasms. No pleomorphism and only scattered mitotic figures of normal configuration were present. Immunohistochemistry demonstrated that the lesional cells were diffuse positivity for CD99 (Figure 5H), with multifocal staining for EMA and focal staining for SATB2. The stain for NKX2.2 was negative. Fusion analysis revealed *FUS::NFATC2* (case #2; Figure 3B) and *EWSR1::NFATC2* (case #3) rearrangements. FISH using a *EWSR1* break-apart probe revealed a significant number of cells with break-apart (split) signal (>20% of lesional cells); however, no *EWSR1* amplification was present (Figure 5H inset). CNV analysis showed a simple karyotype without any copy number alterations (Supplementary Figure S1B,C).

The femoral lesion was resected en bloc and reconstructed with a prosthesis. Both patients are in close follow-up, and no recurrences have developed after 12 and 10 months of follow-up. In case #3, follow-up radiographs show reactive cortical thickening and bone remodeling of the defect (Figure 5D).

### 2.3. EWSR1::NFATC2-Rearranged Soft Tissue Sarcoma (Case #4)

**Figure 6:**
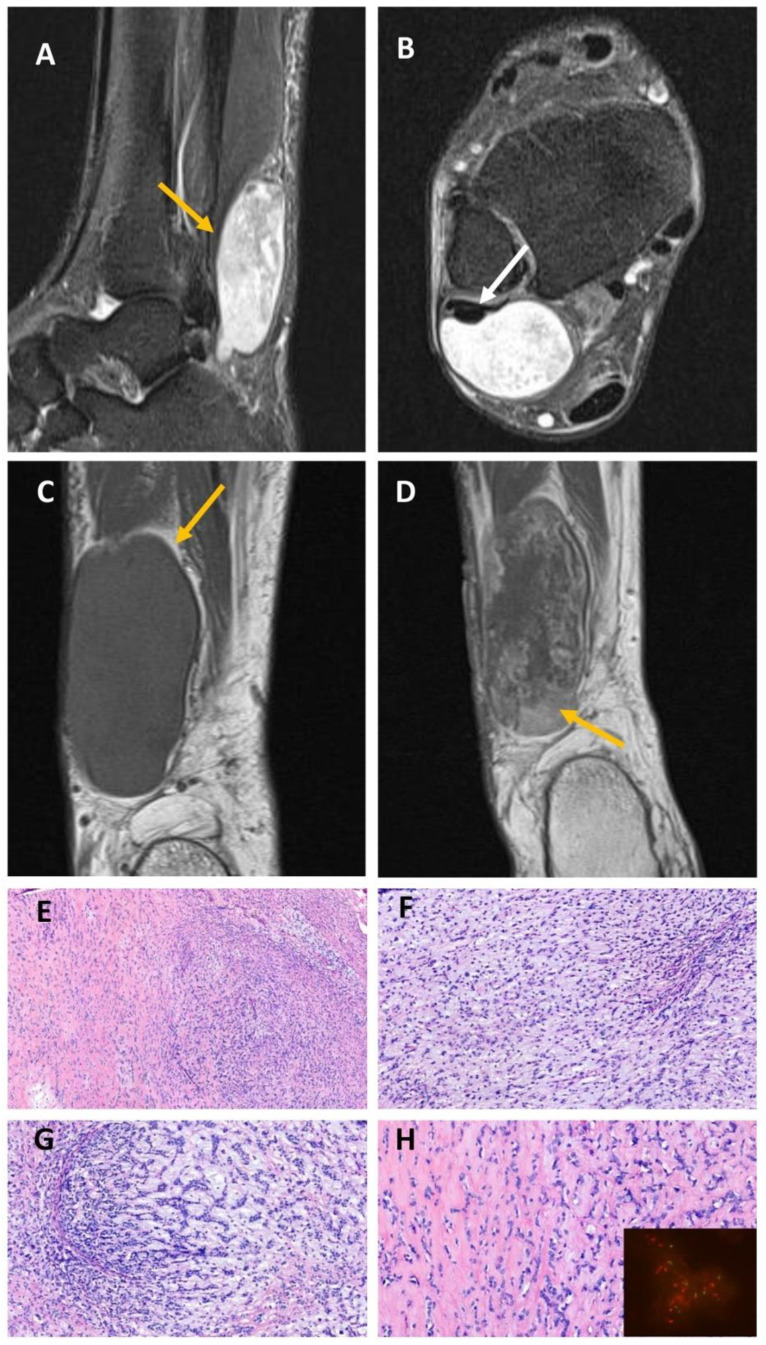
Soft tissue tumor of the distal lower leg in a 69-year-old patient with a *EWSR1::NFATC2* fusion (case #4). (**A**–**D**) MRI images show a well-demarcated lesion in the distal fibularis brevis muscle with a heterogeneous high signal on TIRM (turbo inversion recovery magnitude) images ((**A**)—yellow arrow, (**B**)—white arrow) and homogenous intermediate signal in comparison to the surrounding muscle on T1-weighted image ((**C**), yellow arrow). Heterogeneous contrast enhancement of the lesion (yellow arrow) is seen on the postcontrast-T1-weighted image (**D**). (**E**–**H**) Histologically, the tumor has hypo and hypercellular areas with a variable stroma component—fibrous, myxoid, and myxohyaline. The monomorphic ovoid to epithelioid tumor cells are arranged in interconnecting cords, strands, or nests. (**H**) FISH with *EWSR1* probe shows *EWSR1* amplification (inset).

A 69-year-old male patient complained of a slowly growing mass in the right lower leg. MRI showed a well-demarcated intramuscular lesion in the distal fibularis brevis muscle with peripheral contrast enhancement (Figure 6A–D). The tumor was resected with wide margins. Microscopically, the tumor was heterogeneous in appearance. In the hypocellular areas, it was composed of a myxohyaline or cartilaginous stroma-rich component that contained monomorphic ovoid to epithelioid tumor cells arranged in interconnecting cords and nests adjacent to a hypercellular component. The tumor cells in the hypercellular foci consisted of atypical spindle and round cells arranged in packed cords, nests, and solid sheets (Figure 6E–H). Focally, metaplastic bone was present. Mitotic activity was limited (<5 mitosis/10 HPF), and areas of necrosis were not present. Immunohistochemistry showed cytoplasmic and membranous staining with CD99, and EMA was focally positive. NGS revealed *EWSR1::NFATC2* fusion (Figure 3C). FISH showed break apart of *EWSR1* and *EWSR1* gene amplification (Figure 6H inset). CNV analysis detected gains for chromosomes 22q1.1-13.2 and 22q1.23-2.2 and losses of chromosomes 22p1.21-1.23 and 22q2.2-3.33 (Supplementary Figure S1D).

After 24 months of follow-up, there was no evidence of disease.

### 2.4. EWSR1::NFATC2-Rearranged Bone Sarcoma (Case #5)

**Figure 7:**
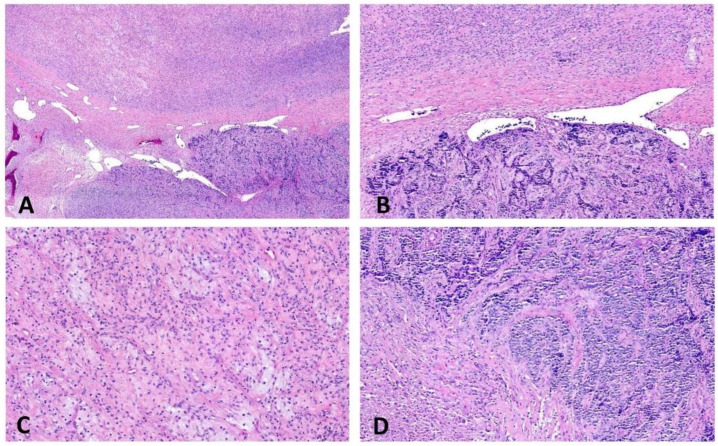
Histology of a high-grade bone sarcoma with *EWSR1::NFATC2* fusion (case #5). (**A**,**B**) Tumor is composed of low- (upper part) and high-grade (lower part) areas. (**C**) Low-grade component consists of cords of monomorphic round to epithelioid cells embedded in a myxohyaline stroma. (**D**) High-grade component is hypercellular and composed of anastomosing cords and trabeculae of highly atypical round cells embedded in a collagenous matrix with area of necrosis.

A 39-year-old-male patient had a femoral tumor composed of a hypocellular low-grade component and high-grade areas with necrosis and significant nuclear atypia (Figure 7; for further clinicopathological details see publication Diaz-Perez et al. [8]). Molecular analysis showed *EWSR1::NFATC2* and *ACTN2::ALK* fusions. CNV analysis of both morphologically distinct areas was performed. The low-grade component had gains in chromosomes 20p, 20q11.1-13.13, and 22q11.21-12.1 (Supplementary Figure S1E), and the high-grade component showed a more complex genomic profile with additional gains of chromosomes 2, 5, 10, 12, 15q23-26.3, 20q11.1-11.22, and 20q13.11-13.13, and losses of chromosomes q21.11-22.33 and 20q12 (Supplementary Figure S1F).

## 3. Discussion

Numerous investigations have documented the increasing promiscuity of gene fusions in bone and soft tissue neoplasms. Accordingly, a precise accurate diagnosis requires correlation of the radiology, morphology, immunohistochemical, and genetic findings.

In our study, we analyzed a biological spectrum of bone and soft tissue *EWSR1/FUS::NFATC2*-rearranged neoplasms. Clinical findings were similar to those previously reported [5,8,9]. SBC and related complex cystic lesions occurred in children in the first two decades, and round-cell sarcomas developed in adults. All tumors occurred in males and ranged in size from 2 to 7.6 cm. In three cases, the long bones were affected. The *EWSR1::NFATC2* bone and soft tissue sarcomas presented as slowly growing masses. This phenomenon is described in several cases of *EWSR1/FUS::NFATC2*-rearranged sarcomas in which symptoms were present for years before diagnosis, suggesting that a pre-existing low-grade component may have undergone biological progression in a subset of patients [8,12].

The histological appearance of the complex cystic bone lesions seems different from that of the wall of a “classic” SBC. In our two complex bone cysts, cellular areas of the monotonous spindle to ovoid cells admixed with abundant foamy macrophages were seen. Recently, Pizem et al., however, described two cases with *EWSR1::NFATC2* fusion with features of a classic SBC next to more cellular areas with admixed histiocytes [5]. Frequently, prominent foamy histiocytes are found in non-ossifying fibromas; however, these lesions present with characteristic radiologic findings and *KRAS* mutation. Moreover, in case #5, as previously described by Diaz-Perez et al. [8], a hypocellular bland spindle cell component with bland morphology was found, suggesting a benign precursor lesion in a tumor with a clear morphologic transition into a hypercellular clearly malignant tumor component. Similar findings were described in two tumors showing a less cellular and more myxoid tumor component, juxtaposed to a hypercellular component with depletion of a matrix, indicating a morphologic spectrum or histologic progression from a low-grade to high-grade histology [10]. Immunohistochemistry with CD99 and EMA plays a limited role, as both benign and malignant *EWSR1/FUS::NFATC2*-rearranged neoplasms can stain positive [5,8]. Diffuse nuclear NKX2.2 staining, however, is observed only in sarcoma and can be useful in a distinction from benign tumors [4,10]. Our complex cystic lesions showed a diffusely positive staining with CD99, a multifocal staining with EMA, and focal staining with SATB2, whereas NKX2.2 was negative.

CNV analysis provides valuable insights into tumor biology. Analyzing chromosomal abnormalities and genomic instability is important in identifying potential prognostic and predictive biomarkers. In our cases of an SBC and two complex cystic lesions, no genomic alterations using CNV analysis and FISH for *EWSR1* amplification were identified. On the other hand, *FUS::NFATC2*-translocated sarcomas exhibited very complex genomic profiles, while in those cases harboring *ESWR1::NFATC2* fusion, fewer genomic alterations were found [6,7,13]. The CNV analysis of the low-grade and high-grade components in case #5, interestingly, revealed additional chromosomal changes in the latter component. It is conceivable that the *EWSR1/FUS::NFATC2* fusion might be a very early event in tumor initiation, and additional events are required for progression.

Significant differences in morphology, transcriptional profile, and behavior are found when comparing sarcomas harboring *EWSR1::NFATC2* and *FUS::NFATC2* fusions [14]. The latter is more aggressive and transcriptionally resembles *CIC*-rearranged sarcomas. Additionally, recurrent fusion gene amplification is almost exclusively detected in the *EWSR1::NFATC2*-rearranged sarcomas. The other benign or malignant tumor types harboring *FUS* or *EWSR1* translocation show no gene amplification using FISH [8,9,10]. Higher expression levels of fusion transcripts found in *EWSR1::NFATC2*-rearranged sarcomas could also be an explanation for the malignant behavior [3].

## 4. Materials and Methods

### 4.1. Patients and Tumor Characteristics

Five *EWSR1/FUS::NFATC2*-rearranged neoplasms, including one simple bone cyst, one soft tissue round cell sarcoma, and two complex cystic bone lesions diagnosed at the D&R Institute of Pathology, Medical University of Graz, from 2018–2022, and one round cell bone sarcoma previously reported in 2019 [8], were analyzed. Institutional ethical approval was obtained from the Ethics Committee of Medical University of Graz. All cases were reviewed by two bone and soft tissue pathologists (I.B. and B.L.-A.).

### 4.2. Immunohistochemical Analysis

From each FFPE TMA block, three 4 µm thick sections were cut. Immunohistochemistry was performed on the Benchmark Ultra platform (Ventana Medical Systems, Tucson, AZ, USA) with iVIEW DAB Detection Kit (Ventana Medical Systems) using two different assays. Two antibodies with appropriate on-slide positive controls were used: EMA mouse monoclonal antibody (clone E29; Ventana/Roche, RTU), CD99 rabbit monoclonal antibody (clone 12E7; Dako/Agilent, Denmark; RTU), SATB2 (clone EP281, Cell Marque, The Hague, The Netherlands; 1:100), and NKX2.2 (polyclonal, Sigma-Aldrich, St. Luis, MO, USA; 1:100). Specific cytoplasmic and/or membranous as well as nuclear, moderate-to-strong staining pattern (depending on the antibody) in >1% of tumor cells was considered positive.

### 4.3. Fluorescence In Situ Hybridization

Dual-color, break-apart FISH assay to detect *EWSR1* rearrangement was performed following the manufacturer’s instructions. Briefly, 4 µm thick sections were cut from FFPE blocks, placed on positively charged slides, dewaxed, and rehydrated. The slides were incubated with 10µL of SPEC *EWSR1* probes (Chr22q12.1-q12.2 proximal to the *EWSR1* break-apart region, red; Chr22q12.2, distal, green; prod. Nr. Z-2096-50, Zytovision). Specimens were covered with a coverslip, sealed with rubber cement (e.g., Fixogum), and denaturized for 10 min at 75 °C using a hot plate or hybridizer. After overnight hybridization at 37 °C, washing and counter-staining using DNA stain 4′,6′-diaminodo-2-phenylindole (DAPI) was performed. Fluorescence microscopy was performed using a Nikon microscope, and images were captured by Infinity Capture App v 6.5.6. (Teledyne Lumenera, Ottawa, ON, Canada).

### 4.4. RNA and DNA Workflow

For each sample, genomic DNA and RNA were extracted from FFPE material (10–12 unstained, 10 µm thick sections). H&E-stained sections from a single representative FFPE block were examined, areas of high tumor content were marked, and microdissection was performed using a needle to enrich for tumor content. RNA and DNA were isolated using the Maxwell RSC FFPE Plus RNA/DNA Purification Kit (AS1440/AS1720, Promega, Thermo Fischer Scientific, Waltham, MA, USA) according to the manufacturer´s instructions. RNA was quantified by the QuantiFluor ONE dsDNA System on the Quantus Fluorometer (both Promega).

### 4.5. Tumor-Associated Copy Number Variation Analysis

50 ng genomic DNA were used to prepare libraries with the NEBNext Fast DNA Fragmentation & Library Prep Set for Ion Torrent (New England Biolabs, Hitchin, UK). To obtain libraries with a length between 250 and 350 bp the products were electrophoresed using the E-Gel SizeSelect 2% Agarosegel and the E-Gel Safe Imager (both Thermo Fischer Scientific). A qPCR of the finished libraries was then made with the CFX C1000 Touch Thermal Cycler and CFX96 Real-Time System (Bio-Rad). Sequencing was done on the Ion S5 System (Instrument: Ion S5 Sequenzer and Server) and Ion Chef Instrument (Thermo Fischer Scientific). About 5,000,000 reads were taken into calculation, visualized and manually evaluated. Ploidy of the sample was calculated via a reference sample of the opposite gender according to the patients.

## 5. Conclusions

*EWSR1/FUS::NFATC2* rearrangements are promiscuous gene fusions found in both benign and malignant bone and soft tissue tumors. The presence of a specific fusion does not determine the diagnosis, clinical behavior, or response to therapy. Therefore, a thorough correlation of radiology, morphology, immunohistochemical profile, and molecular findings is indispensable in rendering the correct diagnosis. It seems that SBCs can present with a broad morphological spectrum ranging from thin- to thick-walled tumors that are hypo to hypercellular with or without numerous foamy histiocytes. Our results indicate that the presence of *EWSR1* gene amplification or gene copy number changes is found only in biologically aggressive neoplasms. In challenging cases, tumors with *EWSR1/FUS::NFATC2* rearrangements can undergo CNV and FISH analysis to help determine the biological potential of the neoplasm and the appropriate treatment and follow-up.

## Figures and Tables

**Table 1:** Clinical, immunohistochemical, and molecular data of all patients.

Case	Age	Gender	Location	Size	Fusion	CD99	EMA	CNV	FISH *EWSR1*	Therapie	Follow-Up (Months)
**1**	17	m	femur diaphysis	3.2 cm	*FUS::NFATC2*	NP	NP	none	NA	curettage	NSD (12)
**2**	10	m	proximal femur	6.7 cm	*FUS::NFATC2*	positive	focal	none	NA	curettage/resection	NSD (12)
**3**	14	m	proximal humerus diaphysis	2.9 cm	*EWSR1::NFATC2*	positive	positive	none	no amplification	curettage	NSD (10)
**4**	69	m	lower leg (distal fibularis brevis muscle)	3.2 cm	*EWSR1::NFATC2*	positive	positive	gain 22q1.1-13.2 loss 22q1.21-1.23 gain 22q1.23-2.2 loss 22q2.2-3.33	amplification	resection	NSD (24)
**5 (low-grade)**	39	m	femur	14.5 cm	*EWSR1::NFATC2*	positive	focal	gain 20p gain 20q11.1-13.13 gain 22q11.21-12.1	amplification	resection	NSD (30)
**5 (high-grade)**								gain 2 gain 5 gain 10 gain 12 gain 15q23-26.3 gain 20q11.1-11.22 gain 20q13.11-13.13 loss q21.11-22.33 loss 20q12			

Legend: m—male; NA—not available; NP—not performed; NSD—no sign of disease.

**Table 2:** Comparison of radiographic characteristics between a simple bone cyst and our two *EWSR1/FUS::NFATC2* cystic bone lesions.

	Simple Bone Cyst	*EWSR1/FUS::NFATc2* Complex Cystic Lesions
Location	central; proximal long bone (humerus, femur)	central; proximal long bone
Osteolysis	geographic	geographic
Border	well defined	well defined
Zone of transition	narrow	narrow
Reactive interface/margin	thin sclerotic margin	surrounding sclerosis discontinuous or absent
Cortical involvement	endosteal scalopping possible	endosteal scalopping
Periosteal reaction	no	no
Soft tissue component	no	yes
Fracture	yes	yes

## Data Availability

Not applicable.

## Supplementary Materials

The following supporting information can be downloaded at: https://www.mdpi.com/article/10.3390/ijms232416196/s1.
