# Supplementary material for: Implementation of Copy Number Variations-Based Diagnostics in Morphologically Challenging EWSR1/FUS::NFATC2 Neoplasms of the Bone and Soft Tissue
Source: Int J Mol Sci. 2022 Dec 19;23(24):16196. doi: 10.3390/ijms232416196 (PMC9784784; doi:10.3390/ijms232416196)
Supplement: Supplementary file 1 [file ijms-23-16196-s001.zip › ijms-2008272-supplementary.pdf]

Supplement Legend

Supplement 1. CNV analysis. A) Simple bone cyst (case #1), B-C) *FUS/EWSR1::NFATC2* rearranged complex cystic bone lesion (case #2 (B) and case #3 (C)), D) high-grade soft tissue sarcoma (case #4), E-F) high-grade bone sarcoma (case #5) (low-grade (E) and high-grade component (F)).

A

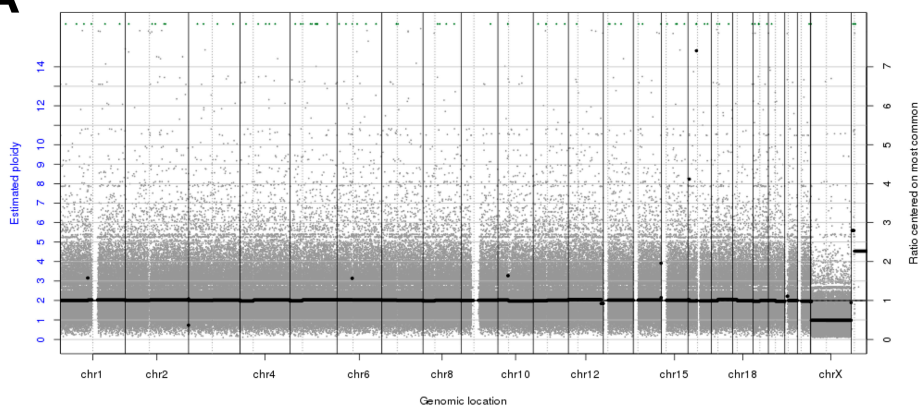

B

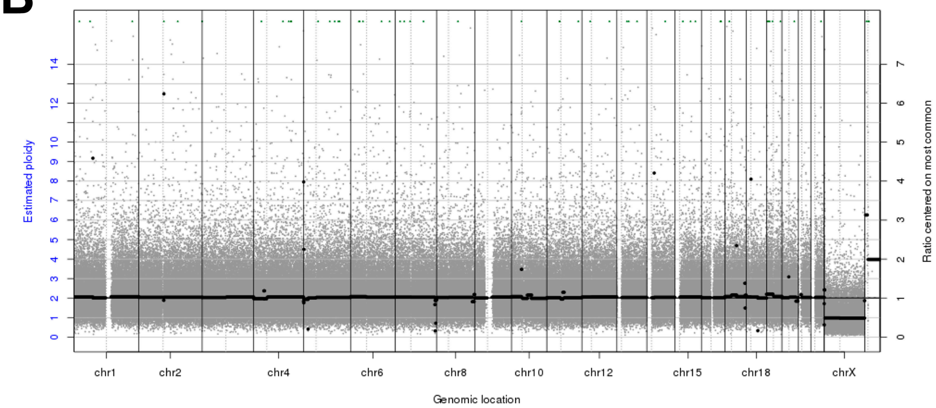

C

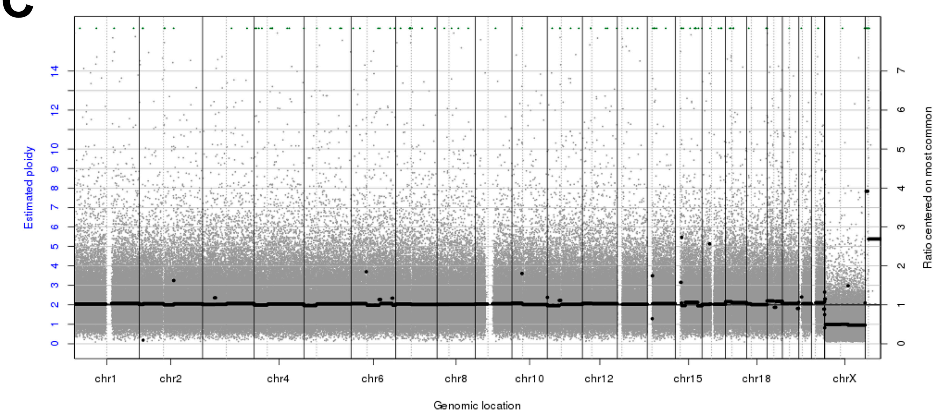

D

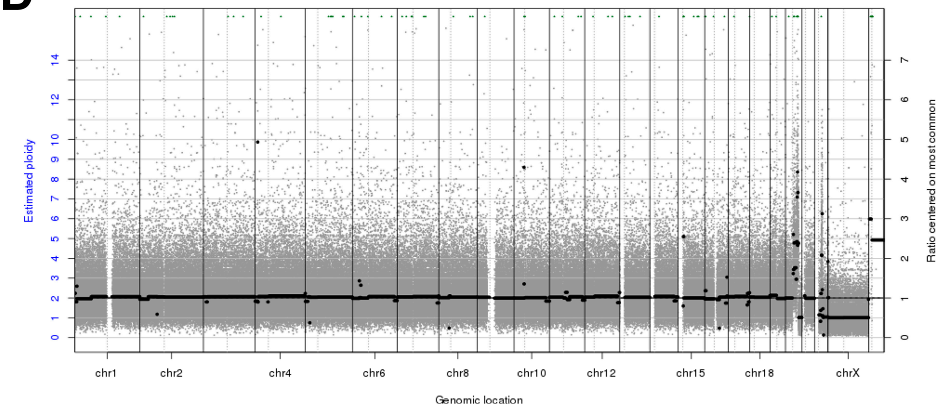
Estimated ploidy
Ratio centered on most common
chr1
chr2
chr4
chr6
chr8
chr10
chr12
chr15
chr18
chrX
Genomic location

E

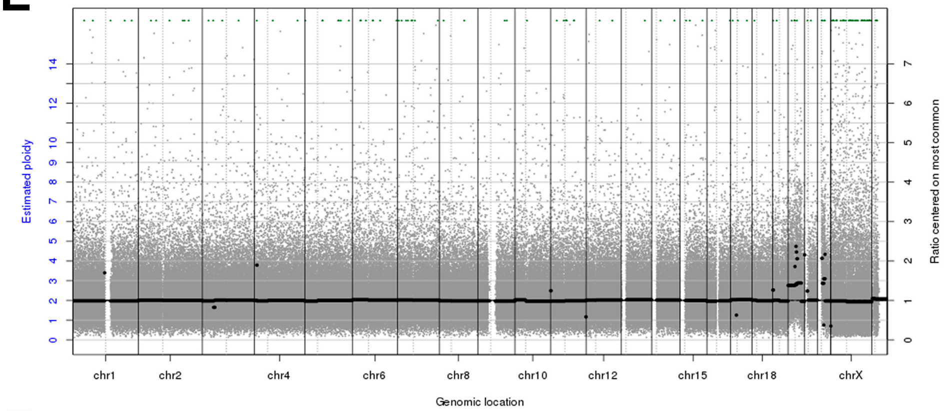
Estimated ploidy
Ratio centered on most common
chr1
chr2
chr4
chr6
chr8
chr10
chr12
chr15
chr18
chrX
Genomic location

F

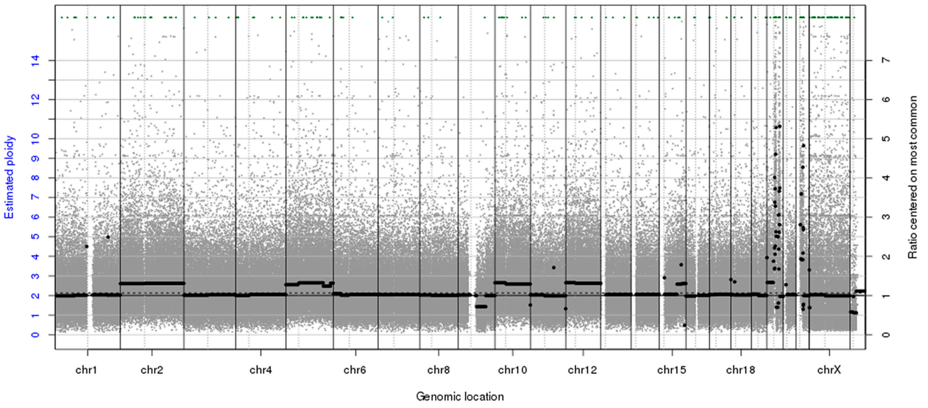
Estimated ploidy
Ratio centered on most common
chr1
chr2
chr4
chr6
chr8
chr10
chr12
chr15
chr18
chrX
Genomic location
